# The Pseudo-Sign of Leser-Trélat: A Rare Presentation

**DOI:** 10.7759/cureus.35155

**Published:** 2023-02-18

**Authors:** Barbara S Suening, Peter J Neidenbach

**Affiliations:** 1 Medicine, Edward Via College of Osteopathic Medicine, Spartanburg, USA; 2 Dermatology, Edward Via College of Osteopathic Medicine, Spartanburg, USA

**Keywords:** internal malignancy, elevated serum tumor markers, eruptive seborrheic keratosis, leser-trélat sign, pseudo-sign of leser-trélat

## Abstract

The true sign of Leser-Trélat is a rare cutaneous marker suggestive of an underlying malignancy. Its hallmark finding is the abrupt onset of multiple seborrheic keratoses (SKs) that increase rapidly in number and/or size within weeks to months. When the ominous finding is present, the associated tumor is usually aggressive and portends a poor prognosis. The “pseudo-sign” of Leser-Trélat also presents with the rapid onset of multiple SKs, but without any underlying disease. It is less well-known, and there are only a few reports documenting the phenomenon. This paper reports the case of an 89-year-old male who presented with multiple SKs that rapidly progressed over his scalp, neck, arms, back, trunk, and legs within two to three weeks. A clinical workup revealed elevated pancreatic tumor markers. His cancer antigen (CA) 19-9 levels were 52 U/mL (normal range 0-37 U/mL). Computed tomography (CT) of his abdomen/pelvis without intravenous (IV) and oral contrast showed no evidence of cancer. Bothersome SKs, such as those where his clothes were rubbed against, were destroyed via cryotherapy or shave removal under local anesthesia depending on their size. The patient has remained without any symptoms or findings of an underlying malignancy, confirming that his presentation was consistent with the pseudo-sign of Leser-Trélat. Because it can be concerning when a patient suddenly develops multiple large SKs, recognition of the pseudo-sign is important to determine the appropriate course of action.

## Introduction

Seborrheic keratoses are common epidermal neoplasms caused by the clonal expansion of immature keratinocytes [1]. They become clinically concerning when there is rapid development or progression, as in the sign of Leser-Trélat. The phenomenon was first described in the 1800s and takes its name from Edmund Leser and Ulysse Trélat who, at the time, were investigating cherry angiomas as a cutaneous marker for underlying malignancies [2]. The actual discovery of explosive seborrheic keratoses and their association with visceral cancer was made by Hollander in the 1900s. To date, there does not exist quantified criteria to diagnose the syndrome [3]. In about 75 percent of reported cases, the lesions were most apparent in the trunk or chest [4]. Other common sites are the extremities, face, and neck. Predominant cancer entities include adenocarcinomas of the gastrointestinal tract, most notably the pancreas, and stomach [5]. In a smaller number of cases, the sign’s association with lymphoproliferative disorders has also been documented. In the setting of the sign of Leser-Trélat, it has been hypothesized that the sudden eruptions are the results of cytokines and growth factors produced by neoplastic cells, and its resolution tends to follow the treatment of the underlying cancer [6,7]. To a lesser extent, the eruption of multiple seborrheic keratoses has also been documented in nonmalignant conditions such as HIV, pregnancy, and cytarabine therapy [8,9].

The pseudo-sign of Leser-Trélat is believed to be less clinically recognized as it is a newer term. Its significance has grown as the validity of the true sign of Leser-Trélat has become debatable [10]. There are increasing reports of the sign of Leser-Trélat presenting without concurrent malignancies despite extensive workup. This challenge has given rise to the “pseudo” sign. It was first defined in 2004 by Patton et al and was described as the exacerbation of pre-existing seborrheic warts in the setting of chemotherapy use [10]. However, in more recent literature, the definition has broadened. In a paper published by Husain et al, the pseudo-sign was described as the eruption of multiple warts without concern for underlying malignancy [11]. For clarification purposes, that is the definition this text has gone by. It is thought to be related to environmental factors, such as sun exposure or aging, rather than an underlying disorder or disease. Workup should include a physical examination and a thorough review of the individual's medical history [11]. Blood tests should be ordered to help rule out the possibility of an underlying internal disorder or disease. CT scans or MRIs may be suggested to help evaluate the individual's overall health and to look for any underlying disorders or diseases [11].

Overall, there is limited text discussing the workup and management for the pseudo-sign of Leser-Trélat. This report supports the existence of the Pseudo-Sign of Leser-Trélat and aims to increase awareness of it, as well as to highlight gaps in the medical literature regarding the phenomenon. While the pseudo-sign of Leser-Trélat is generally less serious than the true sign of Leser-Trélat, awareness of the clinical sign allows for a more accurate plan of care and may decrease the need for extensive or unnecessary malignancy workups.

## Case presentation

**Figure 1:**
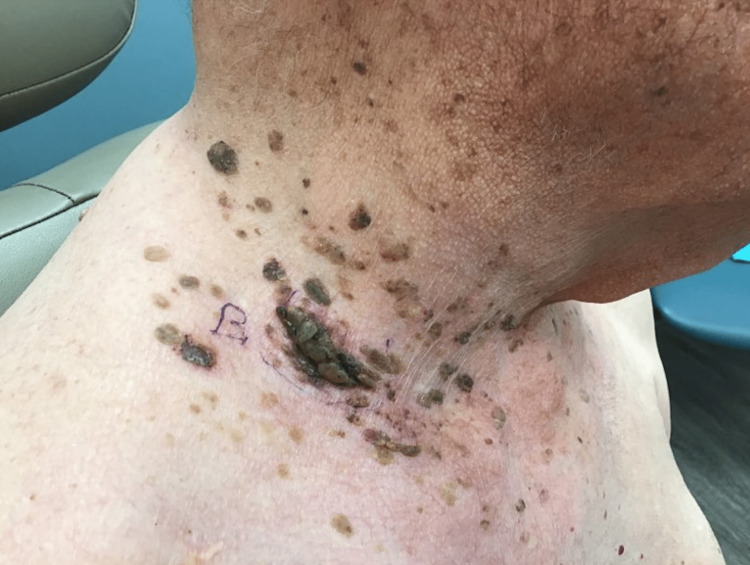
Patient's right side of neck during initial encounter

**Figure 2:**
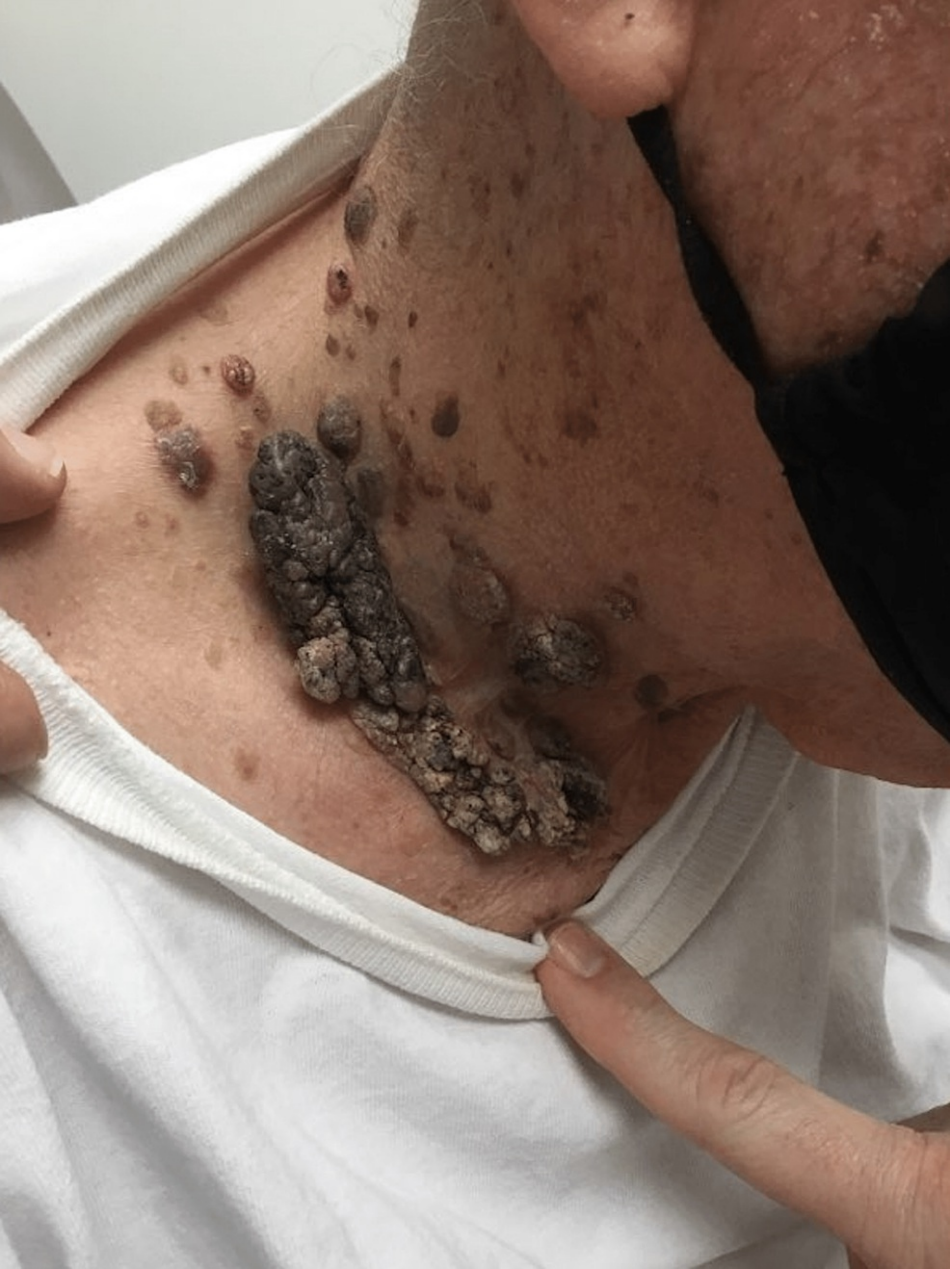
Patient's right side of neck 2-3 weeks after initial encounter

**Figure 3:**
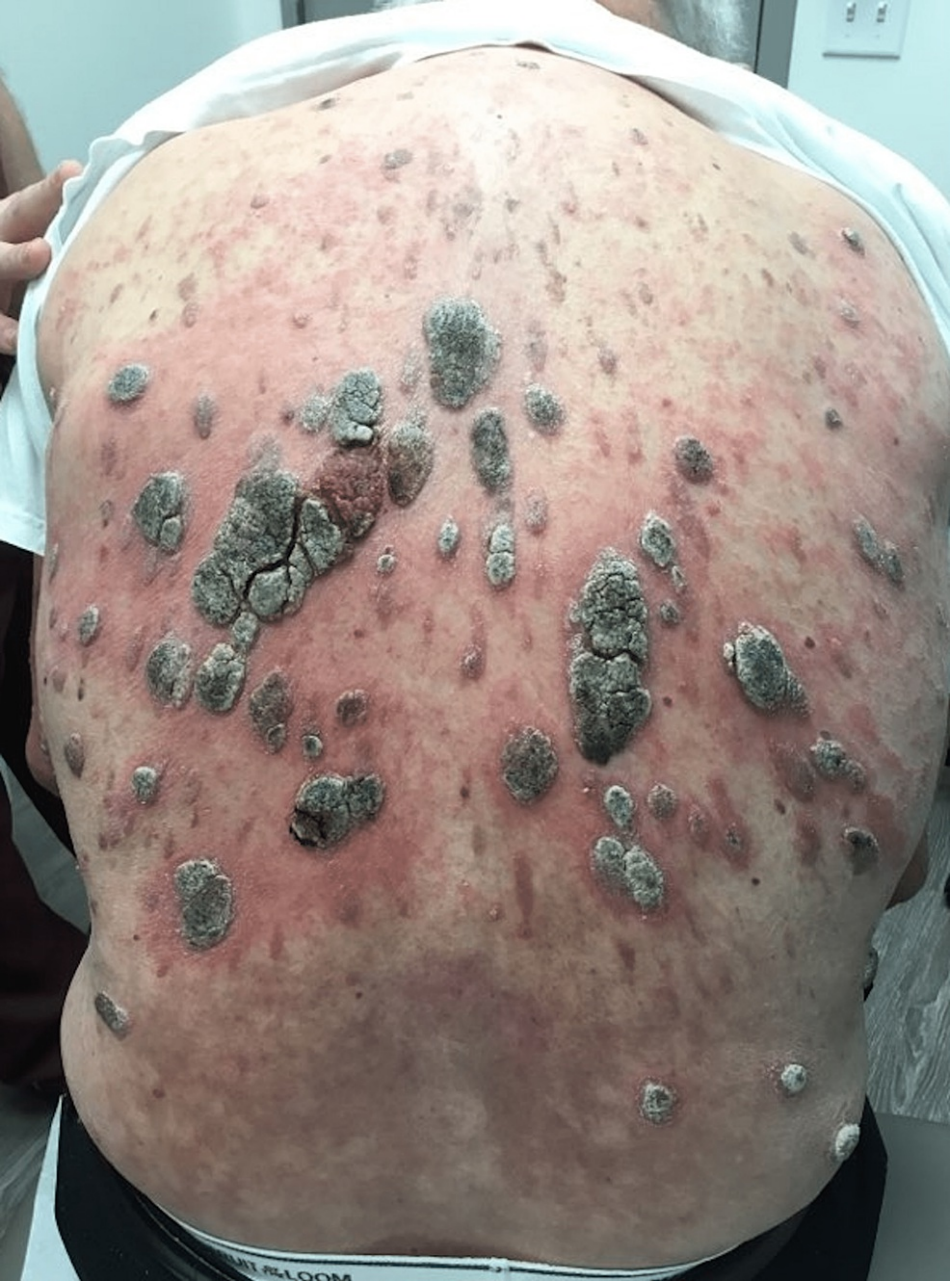
Patient's back during initial encounter

An 89-year-old Caucasian male presented for the evaluation of multiple SKs that progressed suddenly. He had a 40-year history of biopsy-proven SKs but was concerned about their rapid development over the past two to three weeks (Figure 1, 2). The “stuck-on” waxy papules were scattered along his scalp, neck, arms, trunk, back, and legs. The SKs, especially those on his back, were accompanied by surrounding erythema and were mildly pruritic (Figure 3). A review of the systems was negative for abdominal pain, nausea, early satiety, or unexplained weight loss. He denied new onset back pain, fever, rigors, chills, diarrhea, or rectal bleeding. There were no aggravating factors reported.

His past medical history included benign prostatic hyperplasia, chronic kidney disease stage 3B, hypertension, and osteoarthritis. His past surgical history included a cholecystectomy, shoulder surgery, suprapubic prostatectomy, as well as excisions for non-melanoma skin cancer. He denied drinking alcohol, was a former smoker, and was up to date on his vaccinations. He had a normal colonoscopy eight years before his presentation. Medicines included aspirin, atorvastatin, indapamide, lisinopril, metoprolol succinate, and potassium chloride, all without any side effects. He reported an allergic reaction to amlodipine, stating it caused him gingival hyperplasia. He denied a family history of melanoma, breast cancer, and malignant hyperthermia.

Due to suspicion of the sign of Leser-Trélat, a pancreatic tumor profile, complete blood count, and comprehensive metabolic profile were ordered. The results showed elevated cancer antigen (CA) 19-9 levels: 52 U/mL (normal range 0-37 U/mL). Carcinoembryonic antigen (CEA) levels were 4.5 ng/mL. The reference range for CEA in non-smokers is below 3.9 ng/mL and the reference range for CEA levels in smokers is below 5.6 ng/mL. This patient was a former smoker. Due to the elevated CA 19-9 levels, CT of the abdomen/pelvis without IV and oral contrast was recommended by his primary care physician. CBC and CMP were within normal limits.

CT of the abdomen/pelvis without IV and oral contrast revealed unremarkable findings suspicious for cancer. The pancreas was normal in size and its contour was without focal lesions. The CT did identify a vague 9 mm nodule on the left kidney and a 4 cm cyst in the lower pole of the left kidney. It was compared to a prior CT of the abdomen/pelvis without IV and oral contrast from the year beforehand, which revealed unchanged findings. Due to the prevalence of gastric adenocarcinoma as an underlying malignancy in the setting of the sign of Leser-Trélat, consideration was given to performing an upper gastrointestinal endoscopy. The decision was deferred to his gastroenterologist. Since there were no suspicious findings on CT, the invasive procedure was not recommended. No additional imaging or testing was warranted.

Triamcinolone acetonide 0.1% topical cream was prescribed for the relief of itching. Bothersome SKs, such as those where his clothes were rubbed against, were destroyed via cryotherapy. Larger ones were removed by shave removal under local anesthesia. The patient was followed for a year and remained without any evidence of cancer.

## Discussion

Seborrheic keratoses are most prevalent in middle-aged and elderly populations, and present as well-demarcated, “stuck-on” lesions that may progress and thicken over time. They are usually dark brown or black, and have a waxy or scaly appearance. They can appear anywhere on the body, but are most commonly found on the face, chest, and back [12]. At this time, the exact pathogenesis of SKs remains undetermined. There is some evidence to suggest that epidermal growth factors (EGF) may be involved in their development. Studies have shown that SKs express higher levels of EGF receptors compared to normal skin, and that the levels of EGF receptors increase with the size and thickness of SKs [13]. In addition, studies have shown that the growth of SKs can be inhibited by blocking the EGF receptor with medications [13]. Sun exposure and aging are known risk factors for the development of SKs, and individuals who have had long-term sun exposure, such as outdoor workers or those who spend a lot of time in sunny climates, may be more likely to develop SKs [14]. There is some evidence to suggest that the development of multiple SKs may be inherited as an autosomal dominant trait [15]. However, the exact genetic basis for the development of SKs is not fully understood, and further research is needed to understand the role of genetics in the development of SKs. 

The pseudo-sign of Leser-Trélat has been reported on relatively infrequently. In a 2013 literature review, only 12 published cases on the pseudo-sign were identified through the PubMed database [16]. The average age was 61.8 years. In the same review, 109 cases regarding the true sign of Leser-Trélat were identified. In 68% of those patients, eruptive SKs preceded the discovery of malignancy [16]. Eruptions observed after the diagnosis of a malignancy were reported in about 22% of cases. In the remaining 10%, SKs were identified at the same time the cancer was diagnosed. Frequently reported associations included pruritis and, to a lesser extent, acanthosis nigricans [16]. 

Because our patient was 89-years-old and otherwise asymptomatic, his unremarkable malignancy work-up made it difficult to ascertain whether the explosive SKs were related to an occult malignancy or another undetermined reason. He was generally in good health, and signs of cancer such as unexplained weight loss, early satiety, abdominal pain, nausea, or back pain were absent. Although our patient presented with pruritic lesions, it's a fairly non-specific finding. No evidence of acanthosis nigricans was appreciated. His CT of the abdomen/pelvis was non-concerning for suspicious lesions and did not warrant further testing. Although our patient's CA 19-9 levels were elevated (52 U/mL), this tumor marker has been found to be more clinically significant when: it is above 100 U/mL, the patient is symptomatic, and a mass is noted [17]. A CA 19-9 level between 37 and 100 is considered the “grey zone,” where malignancy is still debatable. Elevated tumor markers can be a sign of cancer, but they can also be caused by other factors [17]. Although the association is rare, studies have reported cases of patients with chronic kidney disease (CKD) presenting with elevated tumor markers and no known malignancy [18]. In stage 4 CKD, the glomerular filtration rate (GFR) is between 15 and 29 milliliters per minute. At this stage, kidney damage is severe and may cause symptoms such as swelling, changes in urine output, and fatigue. In advanced CKD it is possible for patients to have difficulty metabolizing certain substances, such as tumor markers, resulting in elevated serum levels [19]. Additionally, the production of cytokines, supporting the inflammatory response, furthers pathological changes in renal parenchyma. This disturbance not only leads to declining metabolism and decreased GFR, but also to impaired excretion of those substances. CA 19-9, CA 15-5, CEA, and HCG are some of which have been reported [20].

## Conclusions

This case report supports the existence of the pseudo-sign of Leser-Trélat: the rapid progression of multiple SKs without an internal malignancy. A thorough history and physical examination, as well as appropriate laboratory and radiological investigation, are necessary for evaluation of the pseudo-sign. This case report additionally highlights current gaps in literature regarding the phenomenon. With further investigation into the pathogenesis of eruptive SKs, patient care may be improved and extensive or unnecessary workups for malignancy may be reduced.
